# The Effect of Cerebrolysin in an Animal Model of Forebrain Ischemic-Reperfusion Injury: New Insights into the Activation of the Keap1/Nrf2/Antioxidant Signaling Pathway

**DOI:** 10.3390/ijms241512080

**Published:** 2023-07-28

**Authors:** Basma H. Marghani, Shaymaa Rezk, Ahmed I. Ateya, Badriyah S. Alotaibi, Basma H. Othman, Samy M. Sayed, Mohammed Ali Alshehri, Mustafa Shukry, Mohamed M. Mansour

**Affiliations:** 1 Department of Physiology, Faculty of Veterinary Medicine, Mansoura University, Mansoura 35516, Egypt; 2 Department of Biochemistry, Physiology, and Pharmacology, Faculty of Veterinary Medicine, King Salman International University, El Tor 46612, Egypt; 3 Department of Cytology and Histology, Faculty of Veterinary Medicine, Mansoura University, Mansoura 35516, Egypt; 4 Department of Husbandry and Development of Animal Wealth, Faculty of Veterinary Medicine, Mansoura University, Mansoura 35516, Egypt; 5 Department of Pharmaceutical Sciences, College of Pharmacy, Princess Nourah bint Abdulrahman University, P.O. Box 84428, Riyadh 11671, Saudi Arabia; 6 Medical Experimental Research Center, Faculty of Medicine, Mansoura University, Mansoura 35516, Egypt; basma_osman_mer22@yahoo.com; 7 Faculty of Agriculture, Cairo University, Giza 12613, Egypt; samy_mahmoud@hotmail.com; 8 Department of Science and Technology, Ranyah University College, Taif University, P.O. Box 11099, Taif 21944, Saudi Arabia; 9 Biology Department, College of Science, University of Tabuk, Tabuk 71491, Saudi Arabia; ma.alshehri@ut.edu.sa; 10 Physiology Department, Faculty of Veterinary Medicine, Kafrelsheikh University, Kafrelsheikh 33516, Egypt; mostafa.ataa@vet.kfs.edu.eg; 11 Department of Anatomy and Embryology, Faculty of Veterinary Medicine, Mansoura University, Mansoura 35516, Egypt

**Keywords:** cerebrolysin, forebrain ischemia-reperfusion injury, neurological deficits, TLRs/NF-kB/cytokines signaling pathway, Keap1/Nrf2/antioxidant signaling pathway, microglia, astrocytes

## Abstract

Forebrain ischemia-reperfusion (IR) injury causes neurological impairments due to decreased cerebral autoregulation, hypoperfusion, and edema in the hours to days following the restoration of spontaneous circulation. This study aimed to examine the protective and/or therapeutic effects of cerebrolysin (CBL) in managing forebrain IR injury and any probable underlying mechanisms. To study the contribution of reperfusion to forebrain injury, we developed a transient dual carotid artery ligation (tDCAL/IR) mouse model. Five equal groups of six BLC57 mice were created: Group 1: control group (no surgery was performed); Group 2: sham surgery (surgery was performed without IR); Group 3: tDCAL/IR (surgery with IR via permanently ligating the left CA and temporarily closing the right CA for 30 min, followed by reperfusion for 72 h); Group 4: CBL + tDCAL/IR (CBL was given intravenously at a 60 mg/kg BW dose 30 min before IR); and Group 5: tDCAL/IR + CBL (CBL was administered i.v. at 60 mg/kg BW three hours after IR). At 72 h following IR, the mice were euthanized. CBL administration 3 h after IR improved neurological functional recovery, enhanced anti-inflammatory and antioxidant activities, alleviated apoptotic neuronal death, and inhibited reactive microglial and astrocyte activation, resulting in neuroprotection after IR injury in the tDCAL/IR + CBL mice group as compared to the other groups. Furthermore, CBL reduced the TLRs/NF-kB/cytokines while activating the Keap1/Nrf2/antioxidant signaling pathway. These results indicate that CBL may improve neurologic function in mice following IR.

## 1. Introduction

The human brain is a highly metabolically active organ. The brain is sensitive to blood flow variations, making up between around 15% (CBF 750 mL/min/CO 5000 mL/min) and only 2.5% of the average person’s body weight (BW) [1]. When the blood flow is disrupted, cerebral ischemia occurs. Neuronal cell loss is the most frequent cause of brain malfunction and neurological abnormalities such as learning, memory, and locomotor limitations [2]. Brain injury results from prolonged episodes of hypotension following surgery or shock [3,4]. Transient cerebral hypoperfusion can occur when the neurohormonal and autonomic mechanisms that control the heart rate and blood pressure are disordered [5].

Brain IR injury is caused by cerebral ischemia and the abrupt restoration of the blood flow [6]. It results in neurological impairments, inflammation, edema, oxidative stress, and cell death [7]. After whole-body ischemia, brain damage occurs because of energy shortage and adenosine triphosphate (ATP) consumption, which alters the ionic balance and causes glutamate release, toxicity, and oxidative stress [8]. Although total-body ischemia is the most common cause of brain dysfunction, reperfusion also causes brain IR injury [9]. Severe neurological defects are caused by severe IR injury [10].

The nuclear factor erythroid 2-related factor 2 (Nrf2)/Keap1 (Kelch-like ECH-associated protein 1) signaling pathway is critical in protecting cells from oxidative damage caused by elevated ROS levels [11]. As a result of ROS activation, Nrf2 produces antioxidant enzymes such as heme oxygenase, catalase, glutathione peroxidase, and superoxide dismutase, protecting cells from the harm caused by oxidative stress [12]. Nrf2 is the principal controller of a wide range of cytoprotective genes in normal physiological circumstances [13]. Reduced brain function following IR injury is not effectively treated with current medicines [11].

CBL is a neuropeptide preparation containing free amino acids and low molecular weight (10 kDa) neuropeptides [12]. It has neurorestorative and neuroprotective properties in primary cultured chicken neurons by lessening excitotoxicity, activating microglia, inhibiting ROS and apoptosis, promoting neuronal sprouting, taking on a neurotrophic role, stimulating neurogenesis, and improving cellular survival in primary cultured chicken neurons [13]. A more recent entry in the Cochrane Database of Systematic Reviews found no/possibly small effect of CBL on preventing all-cause death in acute ischemic stroke [14]. In animal models, CBL administration has been demonstrated to aid in neurological recovery, suggesting that it could cause acute ischemia [15].

According to Barakat et al. [16], CBL injection before focal cerebral ischemia injury reduces cytokine and immune cell infiltration into the ischemic hemisphere. However, its neuroprotective effects in relation to transient global forebrain IR injury have yet to be studied. As a result, we induced a new BLC57 mouse simulation of transient forebrain IR injury via transient dual carotid artery ligation (tDCAL) to assess the functional and neurological deficits after IR injury and to investigate the protective efficacy of CBL 30 min before tDCAL (pre-IR injury) and its therapeutic efficacy 3 h after tDCAL (post-IR injury) on the functional recovery, neuroinflammation, endothelial damage, BBB damage, tissue regeneration and remodeling, and the keap1/Nrf2/antioxidant signaling pathway.

## 2. Results

### 2.1. CBL Improved Motor Recovery in the Forebrain IR Mice Model

**Figure 1:**
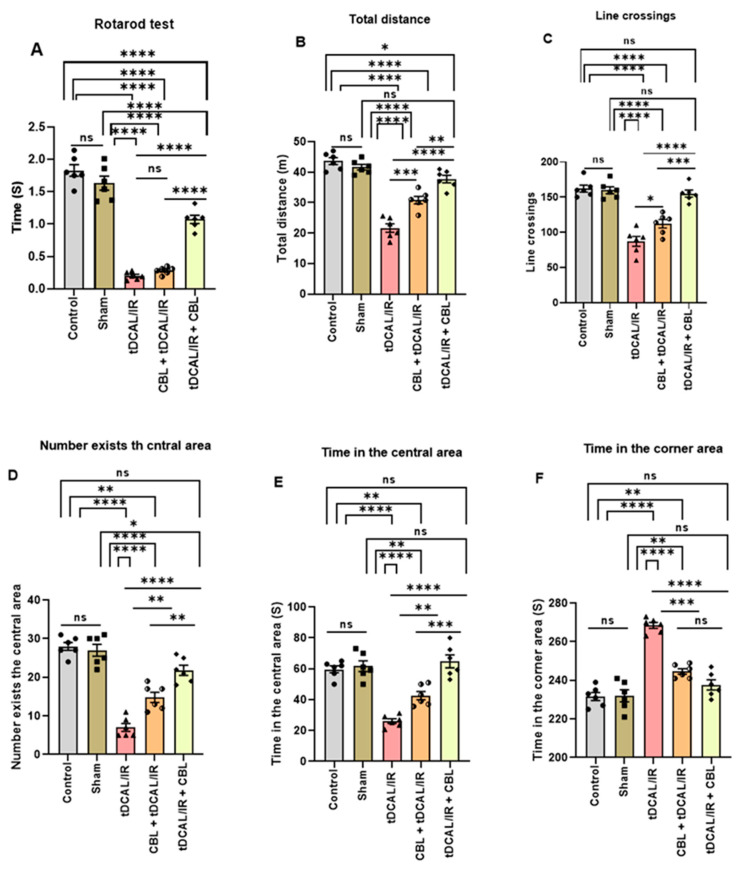
CBL improved neurological functional recovery in the forebrain IR mice model. (**A**) Rotarod test (time, S). (**B**) OFT (total distance, m). (**C**) OFT (line crossings). (**D**) OFT (number exists in the central area). (**E**) OFT (time in the central area, S). (**F**) OFT (time in the corner area, S). Data are shown as mean ± SEM. The data were analyzed using a one-way ANOVA followed by the Bonferroni multiple comparisons test. The asterisks denote a significant difference (* *p* < 0.05, ** *p* < 0.01, *** *p* < 0.001, and **** *p* < 0.0001) between the indicated groups (N = 6).

The results revealed significant (*p* < 0.0001) differences in motor recovery in the IR and CBL-treated mice groups. The IR mice had a substantial decrease in the latency to fall from the rotating rod (0.2000 s) when compared to the control (1.828 s) and sham (1.632 s) mice groups (*p* < 0.0001). However, functional motor recovery was significantly improved in the tDCAL/IR + CBL mice group treated with CBL 3 h after IR, as the animals stayed much longer (1.093 s) on the rotating rod versus the IR group (0.2000 s) (*p* = 0.0002) and the CBL + tDCAL/IR group (0.3000 s) (*p* = 0.0050) (Figure 1A).

The open-field test was used to compare the activity and anxiety levels between the several mouse groups included in the study (Figure 1B–F). In the IR mice, the total distance traveled (21.67 m), the number of line crossings (87.25.), and the number exists in the central area (7.288) were notably (*p <* 0.05) decreased compared to the control (43.65 m, 162.3, and 28.23) group and the sham (41.66 m, 159.8, and 27.13) group (Figure 1B–D). However, the anxiety levels (time in the central area vs. time in the corner area) were significantly higher in the mice following tDCAL/IR (26.12 vs. 268.8 s) compared to the control (59.66 vs. 231.0 s) group and the sham (61.67 vs. 237.0 s) group (Figure 1E,F). The tDCAL/IR + CBL group significantly (*p <* 0.05) increased the total distance, the number of line crossings, and the number that exists in the central area (37.74 m, 155.2, and 21.87) compared to the IR group (21.67 m, 87.75, and 7.28) and the CBL + tDCAL/IR group (30.83 m, 112.20, and 15.17) while decreasing the time in the central area vs. time in the corner area (64.79 vs. 236.7 s) compared to the IR group (26.12 vs. 269.4 s) and the CBL + tDCAL/IR group (42.64 vs. 246.0 s).

### 2.2. CBL Attenuated Neuroinflammation and Enhanced Tissue Regeneration and Remodeling in the Forebrain IR Mice Model

**Figure 2:**
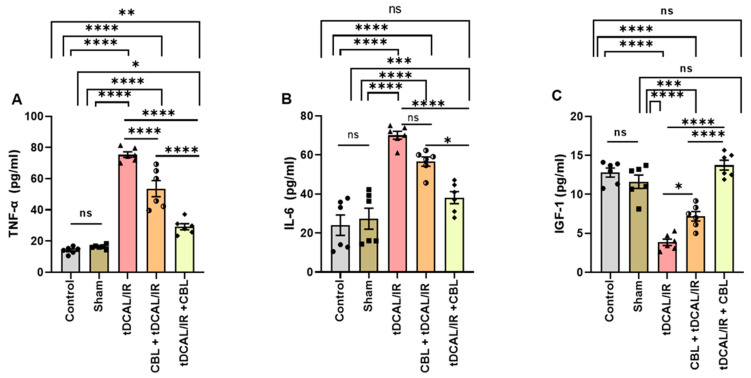
CBL attenuated neuroinflammation and enhanced tissue regeneration and remodeling in the forebrain IR mice model. (**A**) Serum TNF-α level (pg/mL). (**B**) Serum IL-6 level (pg/mL). (**C**) IGF-1 (pg/mL). Data are shown as mean ± SEM. The data were analyzed using a one-way ANOVA followed by the Bonferroni multiple comparisons test. The asterisks denote a significant difference (* *p* < 0.05, ** *p* < 0.01, *** *p* < 0.001, and **** *p* < 0.0001) between the indicated groups (N = 6).

Neuroinflammation complicates the pathologic process of IR injury. We used an ELISA to measure the serum levels of IL-6 and TNF-α and IGF-1 as a neuro-survival factor in the IR mice with and without CBL treatment, as shown in Figure 2. The serum TNF-α (75.38 pg/mL) in the IR mice group was significantly higher 72 h after IR when related to the control (14.06 pg/mL) and sham (16.30 pg/mL) groups (*p <* 0.0001, Figure 2A). In addition, the serum level of IL-6 (70.06 pg/mL) was significantly higher after IR than in the control (34.98 pg/mL) and sham (37.27 pg/mL) groups (*p <* 0.0001 and *p* = 0.0002, Figure 2B). However, the IGF-1 (3.853 pg/mL) levels were meaningfully lesser than in the control (12.80 pg/mL) and sham (11.61 pg/mL) groups (*p <* 0.0001, Figure 2C). CBL administration in the tDCAL/IR + CBL mice group significantly attenuated the IR-induced neuroinflammation and improved tissue repair and remodeling 72 h after tDCAL/IR surgery by decreasing the serum levels of TNF-α (29.15 pg/mL) (*p <* 0.0001) and IL-6 (46.93 pg/mL) (*p* = 0.0008) and increasing the IGF-1 (13.74 pg/mL) (*p <* 0.0001) compared to the IR and CBL + tDCAL/IR mice groups (Figure 2A–C). These findings indicate that transient global forebrain pro-inflammatory cytokine expression increases after IR injury and that neurogenesis is inhibited. CBL treatment 3 h after IR can attenuate neuroinflammation and enhance neuronal repair more than CBL treatment 30 min before IR.

### 2.3. CBL Reduced the Total Brain Water Content in the Forebrain IR Mice Model

**Figure 3:**
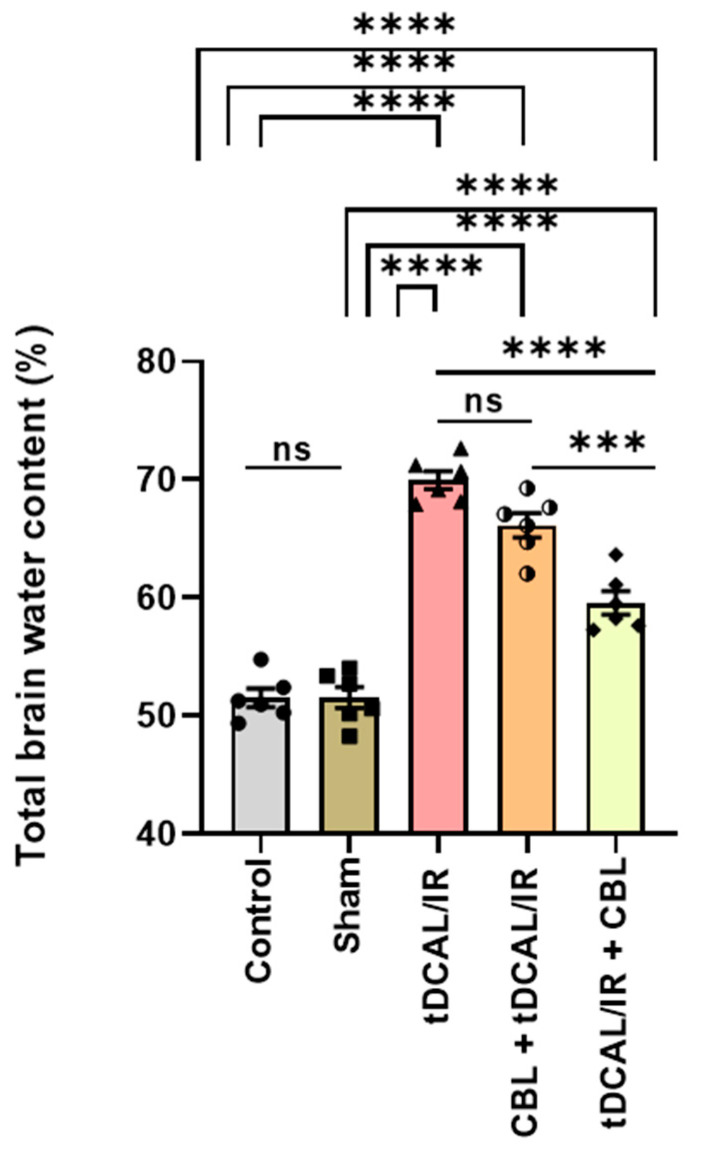
CBL reduced the total brain water content in the forebrain IR mice model—brain water content (%). Data are shown as mean ± SEM. The data were analyzed using a one-way ANOVA followed by the Bonferroni multiple comparisons test. The asterisks denote a significant difference (*** *p* < 0.001, and **** *p* < 0.0001) between the indicated groups (N = 6).

The entire brain water content evaluation in the different experimental groups showed an exceptionally more significant percentage in the IR (69.95%) group compared to the control (51.53%) group and the sham (50.53%) group (*p <* 0.0001). However, the total brain water content was lower (59.57%) in the tDCAL/IR + CBL group 72 h after tDCAL/IR surgery than in the IR group (*p <* 0.0001) and the CBL + tDCAL/IR group (66.15%) (Figure 3).

### 2.4. CBL Repaired the Blood–Brain Barrier Damage in the Forebrain IR Mice Model

**Figure 4:**
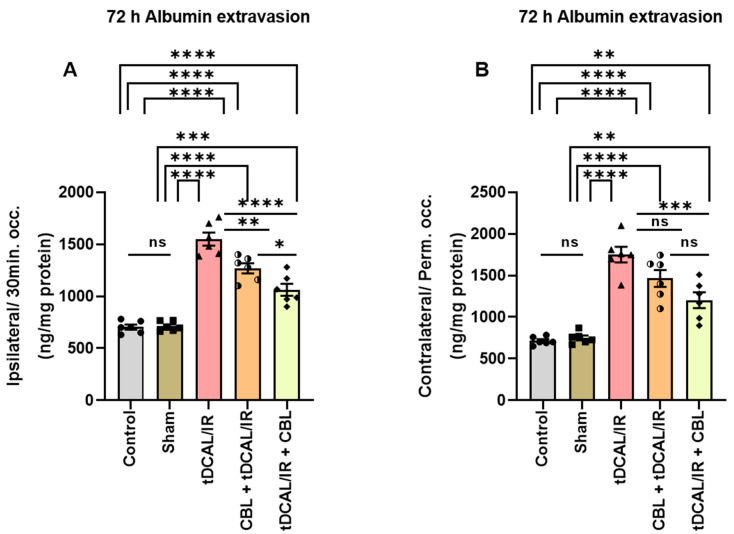
CBL repaired the blood–brain barrier damage in the forebrain IR mice model. (**A**) 72 h albumin extravasation (ipsilateral/30 min. occ., ng/mg protein). (**B**) 72 h albumin extravasation (contralateral/perm. occ., ng/mg protein). Data are shown as mean ± SEM. The data were analyzed using a one-way ANOVA followed by the Bonferroni multiple comparisons test. The asterisks denote a significant difference (* *p* < 0.05, ** *p* < 0.01, *** *p* < 0.001, and **** *p* < 0.0001) between the indicated groups (N = 6).

In this study, the albumin content was more considerably improved in the contralateral/permanent occlusion cerebral hemisphere (1912 ng/mg protein) in the IR group than in the ipsilateral/30 min occlusion hemisphere (1550 ng/mg protein) of the same mice group as compared to the control (722.2 ng/mg protein) group and the sham (725.2 ng/mg protein) group (*p* ≤ 0.0001, Figure 4A,B). However, CBL administration 3 h after IR injury in the tDCAL/IR + CBL group could decrease the amount of albumin extravasation in both the ipsilateral/30 min occlusion (1063 ng/mg protein) and contralateral/permanent occlusion (1414 ng/mg protein) cerebral hemispheres in comparison with the IR group (*p <* 0.0001), the CBL + tDCAL/IR/ipsilateral/30 min occlusion (1269 ng/mg protein), and the CBL + tDCAL/IR/contralateral/permanent occlusion (1734 ng/mg protein, Figure 4A,B). This suggests that the transient forebrain injury induces BBB damage and increased permeability consistent with the greater lesion volume on the side of permanent occlusion and the fact that CBL treatment administered 3 h after IR injury can restore BBB integrity with greater efficacy than CBL administered 30 min before IR.

### 2.5. CBL Mitigated Oxidative Stress in the Forebrain IR Mice Model

**Figure 5:**
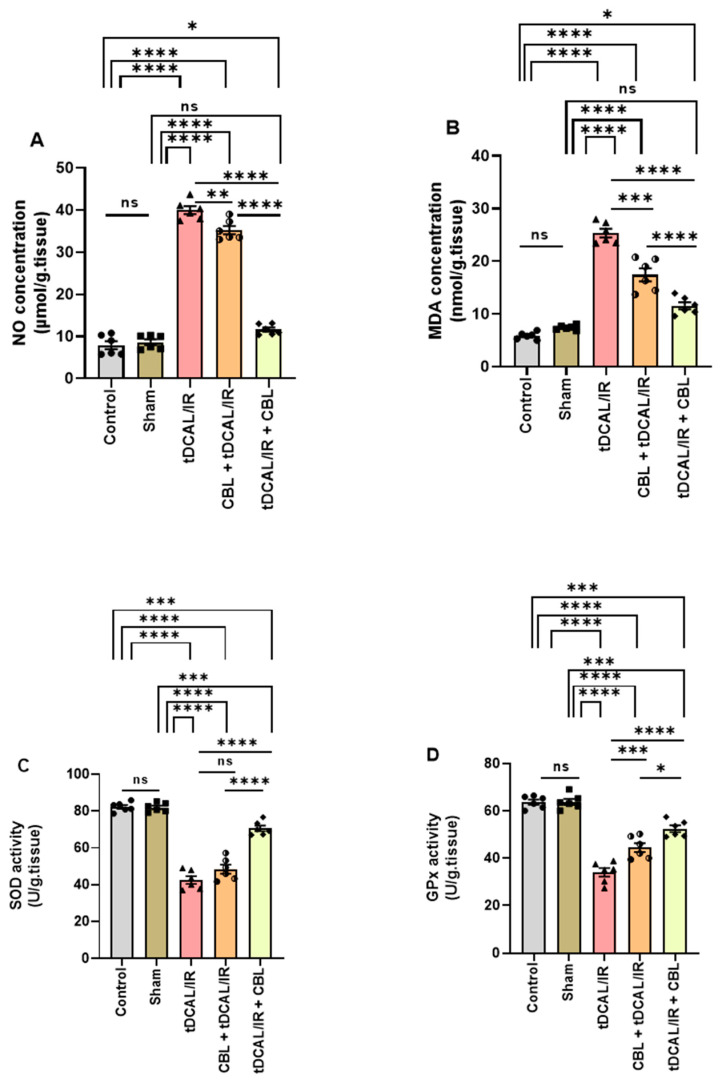
CBL mitigated oxidative stress in the forebrain IR mice model. (**A**) Nitric oxide concentration (NO, contralateral/perm. occ., µmol/g. tissue). (**B**) Malondialdehyde concentration (MDA, contralateral/perm. occ., nmol/g. tissue). (**C**) Superoxide dismutase activity (SOD, contralateral/perm. occ., U/g. tissue). (**D**) Glutathione peroxidase activity (GPx, contralateral/perm. occ., U/g. tissue). Data are shown as mean ± SEM. The data were analyzed using a one-way ANOVA followed by the Bonferroni multiple comparisons test. The asterisks denote a significant difference (* *p* < 0.05, ** *p* < 0.01, *** *p* < 0.001, and **** *p* < 0.0001) between the indicated groups (N = 6).

In contralateral/permanent occlusion cerebral hemisphere homogenates, the NO, MDA, SOD, and GPx activity were evaluated. The IR mice had a significant increase in NO (39.99 µmol/g.tissue) compared to the control (8.053 µmol/g.tissue) and the sham (8.640 µmol/g.tissue) groups (*p <* 0.0001, Figure 5A). The MDA (27.73 nmol/g.tissue) in the IR group was notably higher than in the control (6.008 nmol/g.tissue) and sham (7.395 nmol/g.tissue) groups (*p <* 0.0001, Figure 5B). The SOD (42.57 U/g.tissue) activity was notably reduced in the IR group as compared to the control (82.26 U/g.tissue) and the sham (81.97 U/g.tissue) groups (*p* < 0.0001, Figure 5C). Moreover, the GPX (34.02 U/g.tissue) activity was significantly lower than in the control (63.66 U/g.tissue) and sham (63.79 U/g.tissue) groups (*p* < 0.0001, Figure 5D). CBL administration 3 h after IR in the tDCAL/IR + CBL group resulted in a significant decrease in the NO (11.63 µmol/g.tissue) and MDA (12.41 nmol/g.tissue) concentrations, and an increase in the SOD (70.56 U/g.tissue) as well as GPx (52.41 U/g.tissue) activity as compared to the IR and CBL + DCAL/IR mice groups.

### 2.6. CBL, a Signaling Multi-Target Neuropeptide, Regulated the mRNA Relative Expression of the Target Genes in the Forebrain IR Mice Model

#### 2.6.1. CBL Regulated Neuroinflammation via Inhibiting the TLRs/NF-kB/Cytokines Signaling Pathway

**Figure 6:**
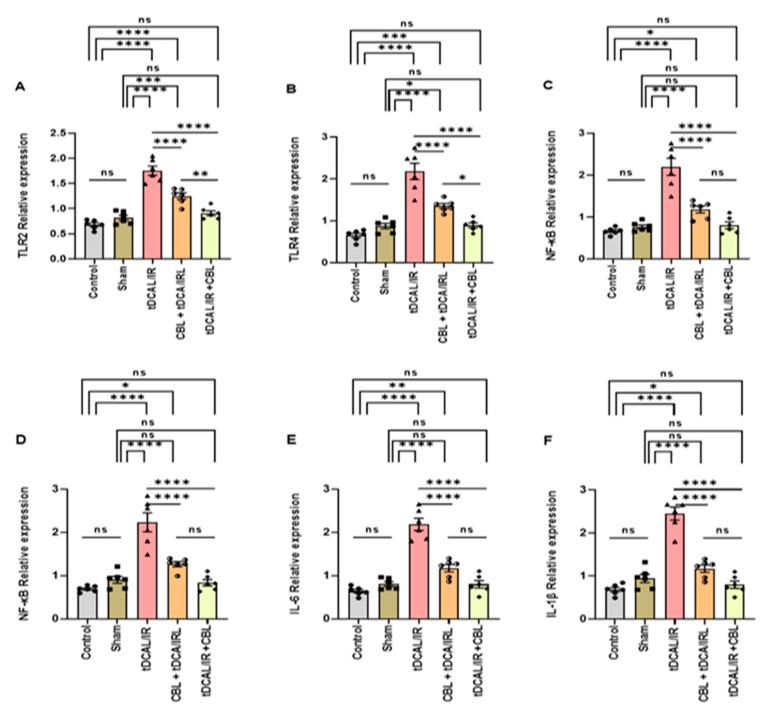

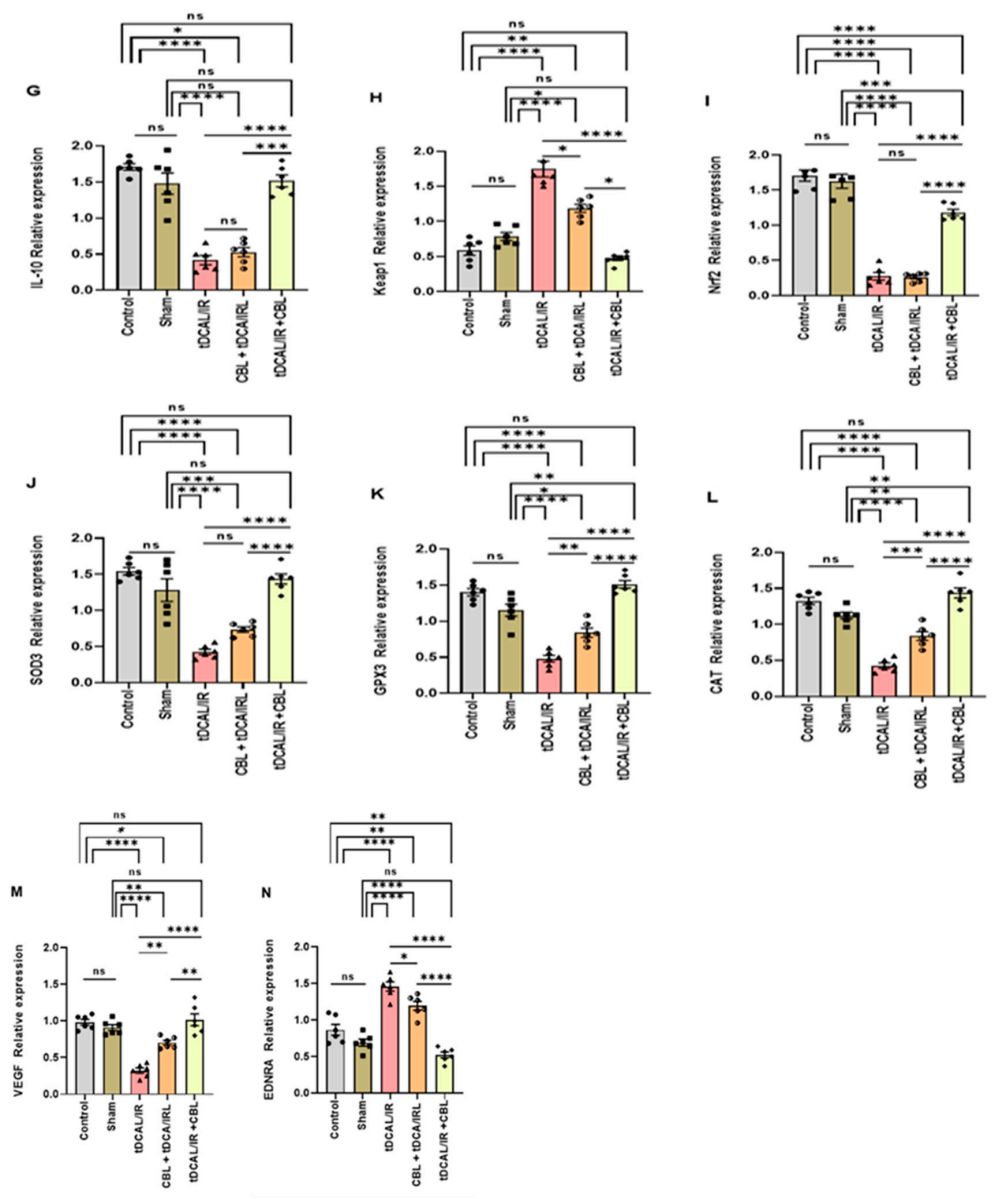
CBL, a signaling multi-target neuropeptide, regulated the mRNA expression of the target genes in the forebrain IR mice model. (**A**) Toll-like receptor 2 (TLR2). (**B**) Toll-like receptor 4 (TLR4). (**C**) Nuclear factor-kappa B (NF-kB). (**D**) Tumor necrosis factor-alpha (TNF-α). (**E**) Interleukin-6 (IL-6). (**F**) Interleukin 1 beta (IL-1β). (**G**) Interleukin-10 (IL-10). (**H**) Kelch-liked ECH-associated protein 1 (Keap1). (**I**) Nuclear factor-erythroid factor 2-related factor 2 (Nrf2). (**J**) Superoxide dismutase 3 (SOD3). (**K**) Glutathione peroxidase 3 (GPX3). (**L**) Catalase (CAT). (**M**) Vascular endothelial growth factor (VEGF). (**N**) Endothelin receptor type A (EDNRA). Data are shown as mean ± SEM. The data were analyzed using a one-way ANOVA followed by the Bonferroni multiple comparisons test. The asterisks denote a significant difference (* *p* < 0.05, ** *p* < 0.01, *** *p* < 0.001, and **** *p* < 0.0001) between the indicated groups (N = 6).

The mRNA expression of TLR2, TLR4, NF-kB, and specific pro/anti-inflammatory (TNF-α, IL-6, and IL-1β/IL-10) genes associated with IR injury was quantified 72 h after IR via RT-qPCR relative to the β-actin reference gene in the contralateral/permeant occlusion cerebral hemisphere in the different experimental groups. In the brain tissues of the IR mice, we discovered that the mRNA expression of TLR2, TLR4, NF-kB, TNF-α, IL-6, and IL-1β was significantly increased (*p* < 0.0001, Figure 6A–F). In contrast, the anti-inflammatory IL-10 gene’s mRNA expression was considerably lower than in the control and sham groups (*p* < 0.0001, *p* < 0.001, Figure 5G). CBL treatment 30 min before IR in the CBL + tDCAL/IR group or three h after IR in the tDCAL/IR + CBL group significantly decreased the TLR2, TLR4, NF-kB, TNF-α, IL-6, and IL-1β *(p <* 0.0001, Figure 6A–F). Still, there was a considerable upsurge in the IL-10 mRNA relative expression in the tDCAL/IR + CBL group (*p* < 0.0001, Figure 6G). CBL administration 3 h after IR was found to be effective in protecting against transient global forebrain ischemia-reperfusion injury via inhibiting the TLRs/NF-kB signaling pathway and neuroinflammation in the forebrain IR mice model, being more effective than CBL administration 30 min before IR.

#### 2.6.2. CBL Regulated Oxidative Stress via Activating the Keap1/Nrf2/Antioxidant Signaling Pathway

At 72 h after IR in the contralateral/permanent occlusion cerebral hemisphere, the mRNA relative expression of Keap1 was significantly upregulated in the IR group compared to the control and sham groups (*p* < 0.0001, *p* < 0.001, Figure 6H). However, the mRNA relative expressions were extensively downregulated and matched to the control and sham groups (Figure 6I–L). CBL treatment 3 h after IR injury (*p* < 0.0001) downregulated the relative expression of the Keap1-mediated upregulation of the Nrf2, SOD3, GPX3, and CAT relative expressions greater than CBL treatment 30 min before IR. These findings indicate that CBL administration after IR was more effective than CBL administration before IR in alleviating IR-induced oxidative injury via activating the Keap1/NrF2/antioxidant signaling pathway in the forebrain IR mice. 

#### 2.6.3. CBL Improved Postischemic Neurovascular Remodeling and BBB Integrity via Upregulating VEGF and Downregulating EDNRA Expression

At 72 h after IR in the contralateral/permanent occlusion cerebral hemisphere, the mRNA relative expression of VEGF was significantly downregulated. In contrast, the EDNRA expression was upregulated in the IR group matched to the control and sham groups (Figure 6M,N). CBL treatment 3 h after IR injury in the tDCAL/IR + CBL group significantly (*p* < 0.05) increased the VEGF relative expression while significantly (*p* < 0.01) decreasing the EDNRA relative expression as compared to the IR and CBL + tDCAL/IR groups. These results illustrate the underlying mechanism by which CBL administration after IR repairs BBB damage and reduces brain edema and vasoconstriction in the forebrain IR mice, including the greater effectiveness than CBL administration before IR.

### 2.7. Histological Findings

The current investigation focused on the internal pyramidal layer of the cerebral cortex, CA2, and dentate gyrus of the hippocampus, striatum, thalamus, and cerebellum because the histological changes after IR injury were more visible in these areas.

#### 2.7.1. CBL Improved Neuronal Survival and Preserved Brain Histoarchitecture in the Forebrain IR Mice Model

**Figure 7:**
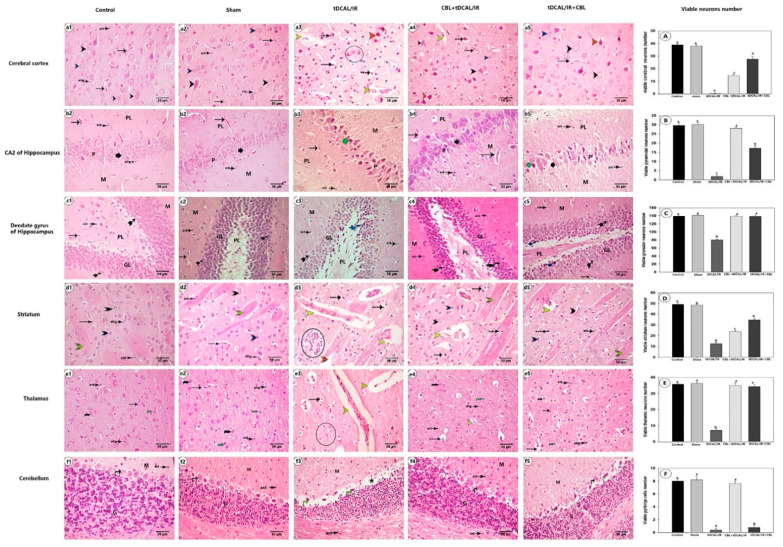
Photomicrograph of a section of mouse brain stained with H&E. 1—Cerebral cortex (**a1**–**a5**) showing survival pyramidal neurons (black arrowhead), survival granular neurons (blue arrowhead), ghost neurons (inside a black circle), and dark eosinophilic neurons (orange arrowhead). 2—CA2 of the hippocampus (**b1**–**b5**) showing a pyramidal survival neuron (short black arrow), dead pyramidal neuron debris (short green arrow), polymorphic layer (PL), pyramidal layer (P), and molecular layer (M). 3—Dentate gyrus of the hippocampus (**c1**–**c5**) showing normal granular neurons (tailed arrow), dark dead granular neurons (blue-tailed arrow), granular ghost neurons (inside orange circular), polymorphic layer (PL), molecular layer (M), and granular layer (GL). 4—Striatum (**d1**–**d5**) showing extensive survival (black triplet arrowhead) or medium-sized neuron (blue triplet arrowhead), intact axonal tract (blue triplet arrowhead), disrupted axonal tract (orange triplet arrowhead), and satellitosis (inside spotted circles). 5—Thalamus (**e1**–**e5**) shows the survival of large- (black curved arrow) or medium-sized (green curved) neurons and ghost neurons (inside a circle). 6—Cerebellum (**f1**–**f5**) showing survival pyriform Purkinje cells (black vertical arrow), debris of dead Purkinje cells (yellow vertical arrow), edematous Purkinje layer (asterisk), granular layer (G), and molecular layer (M). Normal blood vessels (black arrow), perivascular edema (yellow arrowhead), neuronal debris with ample perineuronal space (discontinued arrow), oligodendrocytes (olig), and astrocytes (ast) are all visible. (**A**–**F**) Average neuron percentages in the cerebral cortex (**A**), CA2 (**B**), dentate gurus (**C**), striatum (**D**), thalamus (**E**), and cerebellum (**F**). The data are presented as mean ± SEM. Different letters signify a substantial difference (*p* < 0.05) between the experimental groups.

Brain tissue from the sham and control groups was examined microscopically, revealing that their neurons in the cerebral cortex, CA2, and dentate gyrus of the hippocampus, striatum, thalamus, and cerebellum appeared in the regular organization without any histological alteration, as shown in Figure 7. Even though each zone had distinct and differently shaped neurons, all the neurons in these regions had light-stained (vesicular) nuclei with distinct nucleoli and basophilic cytoplasm. Homogeneous acidophilic neuropil was discovered with dispersed neuroglia cells (astrocytes and microglia) and intact blood vessels between the neuronal cells. In contrast to the control and sham groups, the stained brain section from the IR group demonstrated that diffuse neuronal damage occurred 72 h after reperfusion in these regions, dramatically reducing the number of surviving neurons. The damaged neurons in the cerebral cortex, striatum, thalamus, and cerebellum lost their histoarchitecture forms and were destroyed, resulting in eosinophilic debris distributed throughout the neuropil.

Furthermore, some damaged neurons showed dark eosinophilia, shrinkage with broad perineuronal spaces or dark pyknotic nuclei, or a lack of hematoxylin staining, appearing as ghost cells. The striatal neuropil was found to be vacuolated, with a significant area of perivascular edema, irregular capillary lumen, and an area of satellitosis (many oligodendrocytes surrounded a dead neuron). The administration of CBL either before IR in the CBL + tDCAL/IR group or after IR in the tDCAL/IR + CBL group reduced the incidence of neuropathological changes and dramatically enhanced the number of viable neurons with vesicular nuclei and distinct nucleoli compared to the IR group. When CBL was given after IR in the tDCAL/IR + CBL group, the number of surviving neurons was much more significant in this group than in the CBL + tDCAL/IR group, which was related to the absence of perivascular edema or dead neuronal debris. On the other hand, when CBL was provided before IR in the CBL + tDCAL/IR group, the regular histological organization of the CA2 of the hippocampus and the Purkinje layer of the cerebellum was found.

#### 2.7.2. CBL Retained the Normal Distribution of Nissl Granules within the Brain Neurons in the Forebrain IR Mice Model

**Figure 8:**
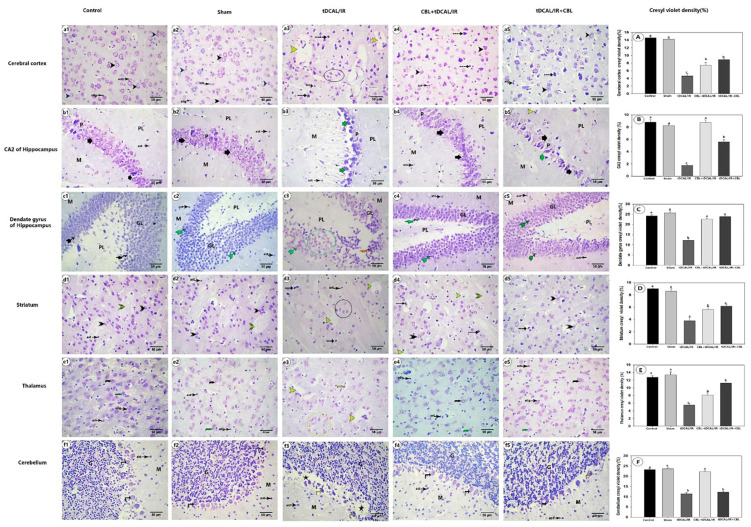
Photomicrograph of a section of mouse brain stained with cresyl violet stain. 1—Cerebral cortex (**a1**–**a5**) showing vesicular nuclei of viable pyramidal (black arrowhead) or granular (blue arrowhead) neurons covered with visible Nissl granules and small faint neurons (inside a black circle). 2—CA2 of the hippocampus (**b1**–**b5**) with basophilic abundant Nissl granule-surrounded vesicular nuclei of pyramidal neuron (black thick short arrow), debris of dark dead pyramidal neuron (green thick short arrow), polymorphic layer (PL), and molecular layer (M). 3—Dentate gyrus of the hippocampus (**c1**–**c5**) showing vesicular nuclei of granular neuron surrounded by Nissl granule (green-tailed arrow), faint and shrunken granular neuron (inside a green circle), and dark dead neuron (orange-tailed arrow), polymorphic layer (PL), molecular layer (M) and granular layer (GL). 4—Striatum (**d1**–**d5**) showing striatum neuron vesicular nuclei surrounded by Nissl granules (dark triplet arrowhead), faint and shrunken striatum neuron (inside a black circle), and axonal tract (yellow triplet arrowhead). 5—Thalamus (**e1**–**e5**) showing vesicular nuclei of viable large (black curved arrow) or small (green curved arrow) neurons capped with visible Nissl granules and small faint neurons (inside yellow circle). 6—Cerebellum (**f1**–**f5**) showing large vesicular nuclei of Purkinje cells surrounded by basophilic Nissl granules (black vertical arrow), dark dead Purkinje cells (yellow vertical arrow), pale ischemic Purkinje layer (asterisk), granular layer (G) and molecular layer (M). Note: neuronal debris with ample perineuronal space (discontinuous arrow), pale ischemic neuropil (yellow arrowhead), oligodendrocytes (olig), and astrocytes (ast). (**A**–**F**) Cresyl violet density as a percentage of the cerebral cortex (**A**), CA2 (**B**), dentate gurus (**C**), striatum (**D**), thalamus (**E**), and cerebellum (**F**). The data are presented as mean ± SEM. Different letters signify a substantial difference (*p* < 0.05) between the experimental groups.

The control and sham groups’ cerebral cortex, hippocampus (CA2 + dentategyrus), striatum, thalamus, and cerebellum had numerous survival and healthy neurons, as shown in Figure 8. The Nissl granules in their cytoplasm were visible and evenly dispersed around their nuclei, preserving the histological architecture. Matched to the control and sham groups, the neurons in these regions were dark with clumped Nissl granules or shrunken with a considerable decline in the amount of Nissl granules (chromatolysis). In most of the neurons in the CBL + tDCAL/IR and tDCAL/IR + CBL groups, the typical distribution of Nissl granules was redetected and their intensity was substantially higher than in the IR group.

#### 2.7.3. CBL Attenuated GFAP Immune Expression in the Forebrain IR Mice Model

**Figure 9:**
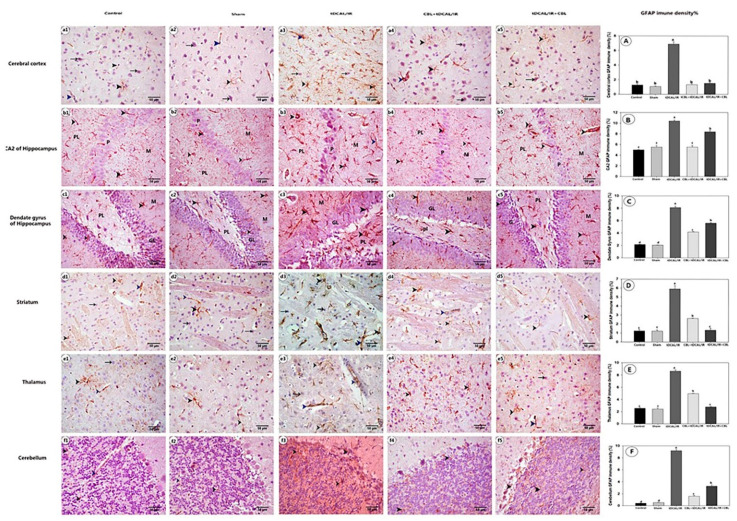
Photomicrograph of the immunohistochemical staining of brain tissue for GFAP. 1—Cerebral cortex (**a1**–**a5**), 2—CA2 of the hippocampus (**b1**–**b5**), 3—Dentate gyrus of the hippocampus (**c1**–**c5**), 4—Striatum (**d1**–**d5**), 5—Thalamus (**e1**–**e5**), and 6—Cerebellum (**f1**–**f5**) show neuronal cells with negative GFAP immune histochemical expression (black arrow). Astrocytes with positive GFAP immune expression in their cytoplasm and cytoplasmic process (black arrowhead) and perivascular astrocyte feet with positive GFAP immune expression (blue arrowhead). Polymorphic layer (PL), pyramidal layer (p), granular layer (G), and molecular layer (M). The immunohistochemical density of the GFAP protein in the cerebral cortex (**A**), CA2 (**B**), dentate gurus (**C**), striatum (**D**), thalamus (**E**), and cerebellum (**F**) (**A**–**F**). The data are presented as mean ± SEM. Different letters signify a substantial difference (*p* < 0.05) between the experimental groups.

As seen in Figure 9, immunohistochemical staining for GFAP in the cytoplasm of astrocytes and their cytoplasmic processes was positive in the control and sham groups. The astrocytes had a tiny cytoplasmic process that was narrow and short. In contrast to the control and sham groups, the astrocytes in the IR group appeared larger, with many thick and elongated cytoplasmic processes (gemistocytes, astrocytes), and the density percent of the GFAP immune expression was substantially greater. Matched to the IR group, the immunological density of the GFAP was reduced considerably when CBL was administered before IR in the CBL + tDCAL/IR group and/or after IR in the tDCAL/IR + CBL group. In the tDCAL/IR + CBL group, the GFAP had the lowest immunological density in the cerebral cortex and striatum. In contrast, the CBL + tDCAL/IR group had the most reduced expression in the CA2 of the hippocampus and cerebellum.

#### 2.7.4. CBL Reduced CD68 Immune Expression in the Forebrain IR Mice Model

**Figure 10:**
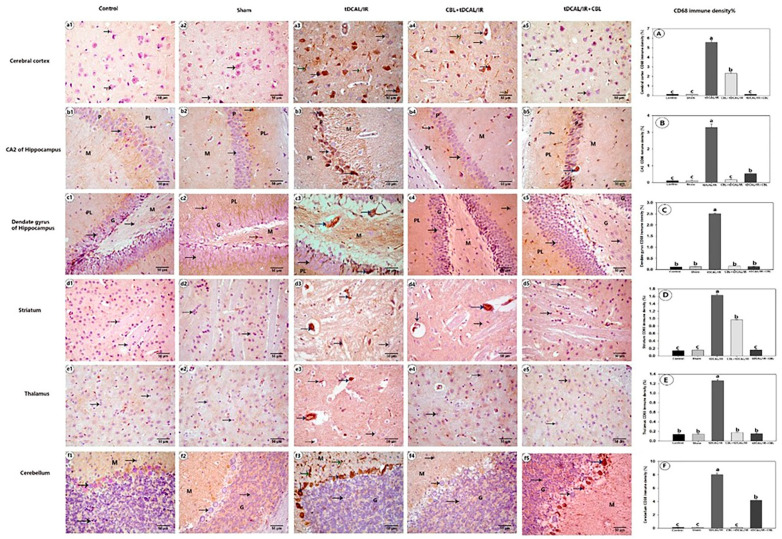
Photomicrograph of enzyme immunohistochemical staining of brain tissue for CD86. 1—Cerebral cortex (**a1**–**a5**), 2—CA2 of the hippocampus (**b1**–**b5**), 3—Dentate gyrus of the hippocampus (**c1**–**c5**), 4—Striatum (**d1**–**d5**), 5—Thalamus (**e1**–**e5**), and 6—Cerebellum (**f1**–**f5**) show neuronal cells with negative CD68 immune histochemical expression (black arrow) and CD68-positive macrophage amoeboid (green arrow) or ramified morphology (blue arrow). Polymorphic layer (PL), pyramidal layer (p), granular layer (G), and molecular layer (M). The immunohistochemical density of the CD86 protein in the cerebral cortex (**A**), CA2 (**B**), dentate gurus (**C**), striatum (**D**), thalamus (**E**), and cerebellum (**F**) (**A**–**F**). The data are presented as mean ± SEM. Different letters signify a substantial difference (*p* < 0.05) between the experimental groups.

As illustrated in Figure 10, the immunological intensity of the CD68-positive macrophages was relatively weak in the control and sham groups. Unlike them, the CD68-positive macrophages in the tDCAL/IR group were stained with CD68 and characterized as amoeboid or ramified. The CD68 immunological density was substantially more significant in the tDCAL/IR group compared with the control and sham groups. When CBL was administered, the CD68 expression was markedly lower in the CBL + tDCAL/IR and tDCAL/IR + CBL groups compared to the tDCAL/IR group.

## 3. Discussion

The forebrain IR model resembles the temporary bilateral common carotid artery ligation (BCCAL) model [17]. Because blood flow is decreased in the frontal, parietal, and temporal lobes of the brain during the ischemic period, although the cerebellum is still perfused without the basilar artery closing, demonstrating a patent and well-established posterior collateral supply, it serves as a model for forebrain IR injury [3]. CBL has been widely used to treat neurological disorders [18]. It is neurotrophic, improves cellular survival, promotes neuronal sprouting, inhibits excitotoxicity, and stimulates neurogenesis, free radical production, microglia, and apoptosis [13].

The current study used behavioral tests to assess the neurobehavioral deficits following tDCAL/IR surgery and evaluate the functional motor recovery with CBL administration at 72 h after IR injury. Our key finding was that the IR group had impaired motor coordination and motor functions, as the mice had decreased latency to fall off the rotating rod, decreased locomotor activity in the open field chamber over 5 min, and an increased anxiety level. The increased anxiety level has previously been confirmed in rodent models of global cerebral ischemia [19] and in humans [20]. Postischemic brain pro-inflammatory microglial activation in the forebrain IR mice model can explain the mice’s neurobehavioral deficits and anxiety-related behavior [21]. Meanwhile, in this study, we found that CBL injection 30 min before IR (CBL + tDCAL/IR) and/or three h after IR (tDCAL/IR + CBL) resulted in substantial advancements in the neurological functional improvement and anxiety 72 h after IR in mice. The fundamental mechanism of CBL’s anti-inflammatory effect, according to Guan et al. [22], is that it inhibits microglial M1 (pro-inflammatory) polarization and promotes microglial M2 (anti-inflammatory) polarization via the CREB/PGC-1α pathway.

In the central nervous system (CNS), microglial cells are the primary pro-inflammatory cells. When brain damage occurs, microglial cells quickly activate, boosting neuroinflammation and oxidative stress and exacerbating delayed neuronal death [23]. Rapid activation of astrocytes and the pro-inflammatory phenotype of microglia during the ischemic first phase leads to the production of pro-inflammatory cytokines (TNF-α, IL-1, IL-6, CCL2, and CXCL10), nitric oxide (NO), and reactive oxygen species (ROS), all of which cause selective neuronal damage [24]. However, the anti-inflammatory phenotype of microglia increases the production of IGF-1, a polypeptide hormone, which contributes to tissue repair and remodeling [25]. The current study discovered that the TNF-α and IL-6 levels in the mice’s serum were notably greater 72 h after IR. Similarly, Jiang et al. [26] found that the pro-inflammatory cytokines were enhanced dramatically in both the serum and the brain after cerebral ischemia and were closely related to the severity of the brain IR injury. Cerebral ischemia can cause damage to neuronal and glial cell membranes, which results in the production of TNF-α, IL-1, and IL-6 [27]. In contrast, CBL injection 30 min before IR (tDCAL + CBL/IR) and/or 3 h after IR (tDCAL + IR/CBL) attenuated IR-induced neuroinflammation by decreasing the TNF-α and IL-6 levels and improving tissue repair and remodeling via increasing the IGF-1 levels in the mice’s serum 72 h after IR injury. IGF-1 reduces neuroinflammation [28] and acts as a survival factor for neurons in vivo [29], and its serum levels correlate positively with clinical outcomes [30,31,32].

In IR injury, acute edema and increased total brain water content occur 30 min following reperfusion [33]. Increased capillary permeability and the permeation of specific proteins from vessels into the tissue cause edema, and oxidative stress induced by IR increases the accumulation of vascular permeability factors like hypoxia-inducible factor (HIF), which causes hyperpermeability via direct action on endothelial cells [34]. Our findings also revealed that the total brain water content was significantly increased 72 h after forebrain IR injury. In addition, after 72 h post-IR damage, BBB permeability was markedly increased, as assessed via albumin extravasation in the brain parenchyma. It rises in IR mice, while albumin extravasation increases considerably in the contralateral/permanent occlusion brain parenchyma. However, CBL administered 3 h after IR injury was found to have a dramatic effect on lowering the brain water content and albumin extravasation in both the ipsilateral/transient occlusion and contralateral/permanent occlusion hemispheres, suggesting its improvement and treatment of postischemic vascular hyperpermeability.

Neuronal mortality caused by IR has been linked to excessive oxidative stress responses, which have been implicated in the pathophysiology of numerous neurological diseases [35]. It may exacerbate brain damage caused by cerebral ischemia [36]. Consistent with the previous study, we found that IR animals had the most significant levels of NO and MDA and the lower activity of SOD and GPx compared to the control and sham groups, indicating that they showed oxidative stress due to IR injury [27]. However, CBL decreased the NO and MDA levels in contralateral/permanent occlusion brain parenchyma while elevating the SOD and GPx activity, which would be presumed to accelerate the scavenging of ROS in the tDCAL/IR + CBL mice compared to the IR mice. Recent studies have demonstrated that CBL is a superior antioxidant and anti-ischemic drug [37].

The current study elucidated the molecular mechanism underlying the potent anti-inflammatory effect of CBL in forebrain IR mice via modulating the TLRs/NF-kB/cytokines signaling pathway. Several innate immune cells include Toll-like receptors, including polymorphonuclear neutrophils, monocytes, macrophages, dendritic cells, and natural killer cells [38]. TLR2 and TLR4 trigger the transcription factor NF-kB and increase the production of inflammatory cytokines [39]. Microglia and astrocytes express a diverse set of TLRs [40]. NF-kB is a transcription factor linked to inflammation, oxidative damage, and apoptosis [40]. Our results showed that the brain tissues of the IR mice showed significantly higher mRNA expression of TLR2, TLR4, NF-kB, TNF-α, IL-6, and IL-1β. In contrast, the anti-inflammatory IL-10 gene’s mRNA expression was considerably lower than in the control and sham groups. CBL administration 30 min before IR (CBL + tDCAL/IR) and/or 3 h after IR (tDCAL/IR + CBL) resulted in 72 h attenuation of IR-induced neuroinflammation via the TLRs/NF-kB/cytokines signaling pathway.

The molecular mechanism underlying the potent antioxidant effect of CBL in transient global IR mice involves modulating the Keap1/Nrf2/antioxidant signaling pathway. It is essential for protecting cells from chemical and oxidative injury by activating antioxidant genes and restoring redox equilibrium [41]. As a result, controlling this pathway is crucial for physiological cellular homeostasis. Keap1 is a key regulator of Nrf2 [42]. As previously stated, in the IR mice, the mRNA expression of Keap1 was substantially higher. However, unlike the control and sham groups, the Nrf2, SOD3, GPX3, and CAT genes were reduced considerably after IR injury.

Meanwhile, CBL treatment before and after IR extensively decreased the expression of Keap1 and upregulated the mRNA expression of the antioxidant genes Nrf2, SOD3, GPX3, and CAT. Here, we signaled that CBL mitigated oxidative stress in the current study, as reflected by the decreasing lipid peroxidation product MDA. In contrast, the increased anti-oxidative enzyme SOD and GPx activities suggest that the neuroprotective effect of CBL might result from reduced IR-induced oxidative stress via the activation of the Keap1/Nrf2/antioxidant signaling pathway. 

Many studies have already established that the primary event after brain ischemia is a cerebral microvascular endothelial function change resulting in a broken blood–brain barrier (BBB) [43]. Furthermore, experimental studies have shown that capillary brain damage can cause more significant perfusion deficits in the ischemic area, resulting in infarction extension [44]. VEGF is a pleiotropic growth factor linked to neurogenesis, axonal plasticity, neuronal survival, vascular permeability, and BBB permeability in IR injury [45], and it is also the primary driver of angiogenesis [46]. According to Stanimirovic et al. [47], endothelin type A may play a role in the etiology of cerebral edema associated with cerebrovascular diseases. The VEGF mRNA relative expression was dramatically downregulated when comparing the IR group to the control and sham groups 72 h after IR in the contralateral/permanent occlusion cerebral hemisphere.

In contrast, the EDNRA expression was significantly elevated. CBL therapy 3 h after IR injury boosted the VEGF relative expression significantly while decreasing the EDNRA relative expression significantly. Interestingly, a recent study [48] discovered that CBL might lessen cerebral edema and pathology in traumatic brain injuries, as well as the blood–brain barrier (BBB) and blood–CSF barrier (BCSFB) permeability abnormalities.

Examination of H&E-stained sections from different brain regions (cerebral cortex, CA2, and dentate gyrus of hippocampus, striatum, thalamus, and cerebellum) of the tDCAL/IR mice showed that the number of survival neurons with vesicular nuclei was meaningfully reduced in this group compared to the control and sham groups. The eosinophilic neuronal debris or injured neurons were scattered in these regions. The expression of transcription factors essential in modulating inflammatory responses and the ROS protein was elevated in patients with cerebral ischemia. Neuronal injury and destruction are brought on by the overproduction of inflammatory molecules, reactive oxygen species (ROS), and oxidative stress [49], and an increase in neuronal death due to apoptosis [50]. Neuronal granules stained with cresyl violet were employed as a structural marker of neuronal survival [51]. The cytoplasm of normal neuronal cells is stained blue by cresyl violet. The ischemic brain sections possess fewer survival and intact neurons than the regular brain sections, so the color is dark blue in a specific region. Still, in the ischemic area, the color is pale blue [52]. In the current study, the density of the cresyl violet stain in the ischemic brain regions in the IR group was reduced, especially compared to the placebo and control groups, which confirmed neuronal death after the transient dual carotid artery ligation.

The histological examination of the brain tissues from the CBL + tDCAL/IR and tDCAL/IR + CBL groups revealed that the administration of the CBL before or after the IR protected the neurons in the cerebral cortex, hippocampus, striatum, thalamus, and cerebellum, as the number of survival neurons in these regions and their cresyl violet stain density were significantly increased compared to the IR group. Neuroprotection requires the early inhibition or reduction of oxidative stress. CBL has been shown in preclinical studies to be neuroprotective and neurotrophic [53], with the potential to decrease the amount of oxidative or cellular stress in the brain [54], generating neuroprotection, which appears to be one of the drug’s most essential effects [55]. Furthermore, overactivated calpain may play a role in destroying cytoskeletal proteins implicated in ischemia pathophysiology. Interestingly, CBL has been shown to inhibit calpain, a Ca2-dependent protease [13]. Furthermore, glutamate toxicity or ischemia is known to disrupt Ca2 homeostasis. Therefore, CBL can protect protein production and prevent neuronal death in many in vivo and in vitro ischemia models by maintaining Ca2 homeostasis [56].

Various molecular signals, like adenosine triphosphate (ATP), cytokines, and others, communicate between microglia and astrocytes [57]. Liddelow and Barres [58] have shown that cytokines released by activated microglia, which are triggered by lipopolysaccharides, can induce reactive astrocytes in vivo and in vitro. Astrocytes respond to brain injury via hypertrophy and the upregulation of GFAP, a hallmark of reactive gliosis in various neurodegenerative diseases [59]. It was found that rapid and intense stimulation of astrocytes results in an inflammatory response and neuronal death [60]. According to the current study, the astrocytes appeared hypertrophied, with long and thick cytoplasmic processes, in the IR group compared with the astrocytes in the control and sham groups. Furthermore, these data were powered by statistical analysis of the GFAP immune density expression as the percent of GFAP immune density meaningfully augmented in the IR group compared to the control and sham groups.

Numerous studies have shown that the lysosomal CD68 protein can be used to stain microglia [61]. High expression is associated with activated phagocytic microglia and macrophages, although low expression is generally associated with resting microglia [62]. When an ischemic stroke occurs, microglia can become activated quickly during the earliest stages of ischemia [63]. Using rat models of ischemic stroke, researchers have found that CD68 expression is a reliable indicator of microglial activation [64]. It was previously believed that activated microglia were the primary inflammatory cells in the central nervous system. This is because they release proinflammatory cytokines, including IL-6, IL-1β, TNF-α, and IFN-γ, as well as reactive oxygen species like nitric oxide via inducible nitric oxide synthase [65].

The density of CD86 immune-stained positive cells was considerably higher in the IR group compared to the control and sham groups, demonstrating microglia activation after IR injury. Conversely, the CD86 and GFAP immunohistochemistry density was significantly reduced in the CBL + tDCAL/IR and tDCAL/IR + CBL groups compared to the tDCAL/IR group. This is consistent with Guan et al. [22]. They showed that CBL might reduce the gene expression of inflammatory markers such as TNF-, IL-1β, iNOS, and COX-2 and stimulate microglial activation toward an anti-inflammatory phenotype. Likewise, Barakat et al. [66] discovered that administering CBL before or after an ischemic brain injury improves histological outcomes and speeds functional recovery by decreasing the immune cell infiltration and cytokines within the ischemic brain. As Kane et al. showed, the attenuation of astrocytic activation after CBL administration in traumatic brain injury was approved [67].

## 4. Materials and Methods

### 4.1. Drugs and Chemicals

Cerebrolysin (CBL) is a porcine brain-derived proteolytic peptide for acute ischemic stroke, dementia, and traumatic brain injury (Ever Neuro Pharma; Unteach, Austria). The purification of the total RNA from animal cells was carried out with an miRNAs Mini Kit (QIAGEN, Hilden, Germany; Cat. No. 217004). A cDNA Reverse Transcription (RT) Kit was used for the RT of the total RNA single-stranded DNA (cDNA) (Cat. No. 4368814) with Maxima SYBR Green qPCR Mix (2X) (Thermo Scientific, Waltham, MA, USA; Cat. No. K0251) and primers for β-actin and oligonucleotides (Daejeon, Republic of Korea). There were only the purest analytical reagents and substances used.

### 4.2. Mice

Seventy-five BLC57 male mice (20 ± 2 weeks old, weighing 25 ± 3 g) were selected from the Medical Experimental Research Center, Faculty of Medicine, University of Mansoura, Egypt. They were housed in polypropylene cages and assigned to various experimental groups. They were kept in standard environmental conditions throughout the experiment at 20–24 °C, with a 12:12 h L/D cycle, 40–55% relative humidity, and free food and water. Before the investigation, the mice were acclimatized for 7 days. All the experimental mice were managed following the instructions for using and caring for laboratory animals in neuroscience and behavioral research, and the experiments were permitted by the Research Ethics Committee, Faculty of Veterinary Medicine, University of Mansoura, Egypt, under animal protocol code No. R/119. The animals were treated following the National Institutes of Health (NIH) ethical guidelines.

### 4.3. Establishment of a Forebrain IR Mouse Model

A transient dual carotid artery ligation (tDCAL/IR) model was used to mimic a temporary forebrain IR injury [3]. Forty-five mice from the total animals used in the study were subjected to tDCAL/IR surgery, and fifteen mice were subjected to sham surgery under aseptic conditions. Permanent ligation of the left common carotid artery (CCA) and temporary fixation of the right with a microvascular clip for thirty minutes was performed. All the animals except the control mice were anesthetized with ketamine hydrochloride (50 mg/kg, i.m.) and xylazine (5 mg/kg, i.m.). The mice were given atropine sulfate of 0.1 mg/kg, i.m., to prevent respiratory distress. After sterilizing the skin with 80% ethanol, a longitudinal incision was made under the neck, and the neck muscles and vagus nerve were bluntly dissected to reveal the carotid bifurcation. The fat and surrounding connective tissue were removed to ligate the left CCA permanently, and being careful not to tangle up the vagus nerve, a 2–3 cm length of 5–0 silk thread will be wound around it, around 1 cm below where the external carotid artery begins. Fat and connective tissue were removed from the right CCA after a brief rest period to prevent vagus nerve overstimulation. Microvascular clips were used to temporarily close the artery for 30 min, with continuous saline rinsing to avoid dryness of the arterial tissue throughout the surgery, after which it was taken out to help with circulation for 72 h. Muscles and skin were closed in layers. Identical surgical techniques were used on the sham-operated mice, except for arterial occlusion. After the surgical procedures, the mice were allowed to recuperate after anesthetic in warmed cages on a heating pad for at least 3 h to maintain their body temperature. They were monitored every 20 min during that time. They were then returned to their cages for the remaining 72 h and given supportive treatment such as warmth, crushed food, and water.

### 4.4. CBL Treatment

**Figure 11:**
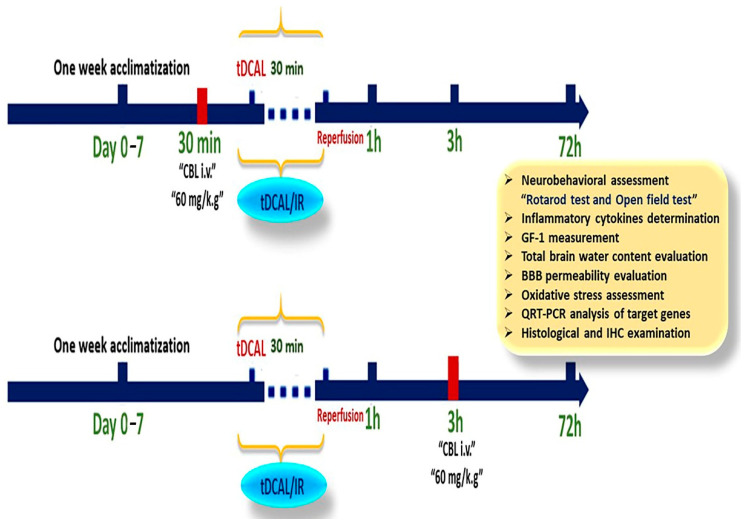
A diagram illustrating the experimental design of cerebrolysin (CBL) treatment 30 min before tDCAL/IR, 3 h after tDCAL/IR, and the assessment of the neuroprotective effects on forebrain ischemia-reperfusion injury.

In this experiment to assess the neuroprotective impacts of CBL on the transient global forebrain IR injury mice model and the underlying mechanisms, 30 mice were selected from the control (15), sham (15), and those subjected to tDCAL/IR surgery (45) and divided into 5 groups (6 mice per group) as follows: Group 1: control group (no surgery was performed); Group 2: sham-operated group (surgery was performed without IR); Group 3: tDCAL/IR group (surgery with IR by permanently ligating the left CA and temporarily closing the right CA for 30 min, followed by reperfusion for 72 h); Group 4: CBL + tDCAL/IR group (CBL was given intravenously at a 60 mg/kg BW dose 30 min before IR) [22]; and Group 5: tDCAL/IR + CBL group (CBL was administered i.v. at 60 mg/kg BW three hours after IR). The mice in the control, sham, and tDCAL/IR groups were administered saline i.v. (experimental design; Figure 11).

### 4.5. Assessment of Neurological–Functional Recovery

In the different experimental groups, the rotarod and open-field behavioral tests assessed neurological–functional recovery (motor coordination, locomotor activity, and anxiety levels) 72 h after tDCAL/IR modeling and CBL treatment.

#### 4.5.1. Rotarod Test

The rotarod test assessed motor function (motor coordination) 72 h after tDCAL/IR surgery with or without CBL treatment [68]. Sticking plasters are applied to a 3 cm diameter by 40 cm long rod to increase its roughness. An electric motor spun the rod at 20 revolutions per minute (rpm). A landing platform with a soft surface was placed 18 cm below the rod to prevent the animals from hurting themselves. The animals were prepared for the test by being trained to carry it out. After resting on the rod for 30 s without movement, a gentle rotation of 4 revolutions per minute was applied. The rats were taught until they could maintain their balance on the rod for at least one minute. After the initial training trial, the rats were placed on the rod for the testing trial, which lasted 300 s and involved the rod rotating at speeds ranging from 4 to 25 revolutions per minute. The time taken to slip off the spinning rod was timed. Motor-impaired mice are more likely to lose their grip on the rod than their healthy counterparts [69].

#### 4.5.2. Open Field Test (OFT)

For assessing the locomotor ability and anxiety-related behavior 72 h after tDCAL/IR surgery with or without CBL treatment [70], a dimly lit square plastic chamber (50 × 50 × 50 cm) held the mice. Individual mice were given 10 min to explore the arena. Each test cleaned the wide field arena with 70% ethanol. The video recorded the open-field behavior for 5 min. Since each animal was positioned in the center, in a single square, it was referred to as a line crossing if it left or crossed a line with its four paws. Open-field activity was captured on camera for five minutes. The total distance traveled by each mouse and the number of lines crossed by its four paws provide information on the animal’s overall locomotor activity. A greater distance and number of line crossings indicate higher locomotor ability. On the other hand, the amount of time the animals spent in the middle and corners of an open field was been examined to ascertain how anxious they were. Their anxiety level increased as they spent less time in the open field’s middle and more in its corners [71].

### 4.6. Euthanasia, Blood, and Tissue Harvesting

At 72 h after surgery and/or CBL treatment, all the mice were starved overnight after the neurobehavioral tests. After being deeply anesthetized with xylazine and ketamine (10 mg/kg and 70 mg/kg, respectively), blood was collected via cardiac puncture and centrifuged at 3000× *g* for 5 min. The serum was kept at −80 °C for further analysis. The animals were sacrificed. The brains were immediately removed from the skull and weighted to obtain the wet weight, then dried and reweighted to calculate the water content of the brain. The brains were bisected on an ice-cold plate to obtain the infarcted ipsilateral cortex/30 min (side of transient occlusion) and the corresponding contralateral cortex/permanent (side of permanent occlusion). Shortly after the brain dissection, 500 mg of each brain tissue was homogenized in a 5 mL phosphate buffer solution (0.01 M), sodium phosphate buffer, pH 7.4, and NaCl (0.14 M) using a homogenizer. The homogenates were centrifuged at 13,000× *g* for 20 min, and the supernatant was collected and stored at −80 °C for analysis of the oxidative stress markers (nitric oxide (NO) and malondialdehyde (MDA)), antioxidant activities (superoxide dismutase (SOD) and glutathione peroxidase (GPX)), and albumin. Parts of the brain tissue were dissected from the contralateral cortex and stored in RNA later (Thermo Fisher Scientific, Brisbane, Australia) for 24 h before being snap-frozen in liquid nitrogen. Other parts of the brain tissue were fixed in 4% paraformaldehyde (PFA) for histological examination and immunohistochemical staining. 

### 4.7. Quantifying the Amount of Water in the Brain

At 72 h following the animal sacrifice, the brains were removed and wet weights were taken. After 24 h in an oven at 120 °C, the samples were dried and reweighed to measure their dry weight. The brain’s water content was then determined using the following formula [72]:

Brain water content % = (Wet weight − Dry weight)/Wet weight × 100

### 4.8. Measurement of Serum TNF-α, IL-6, and IGF-1

The pro-inflammatory cytokines TNF-α and IL-6 serum levels were measured using rodent-specific ELISA kits (Cat. No. 438206, BioLegend, Inc., San Diego, CA, USA and Cat. No. SEA079Ra, Cloud-Clone Corp. Co., Houston, TX, USA). IGF-1 was measured in the serum using a mouse IGF-1 ELISA kit (Cat. No. E-EL-R3001, Elabscience Biotechnology Co., Ltd., Houston, TX, USA), strictly following the manufacturer’s protocol.

### 4.9. Assessment of Blood–Brain Barrier (BBB) Permeability

The extent of the BBB permeability was assessed by quantitating the levels of albumin extracted from the homogenized ipsilateral/30 min occlusion and contralateral/permanent occlusion brain tissue using the commercially available Mouse Albumin ELISA Quantitation kit (Cat. No. ab108792, Abcam, Cambridge, UK) according to the manufacturer’s instructions. The amount of albumin (ng/mg protein) in each sample was calculated from the standard curve.

### 4.10. Quantifying Oxidative Stress and Antioxidant Capacity

To determine oxidative stress-induced neuronal apoptosis after IR injury and/or CBL treatment, the levels of NO, MDA, SOD, and GP_X_ were determined in homogenized contralateral/permanent occlusion brain tissue via the enzymatic colorimetric method. A commercial kit (A012; Nanjing Jiancheng Biological Co., Nanjing, China) determined the NO level. Ready-made assay kits (Bio Diagnostic Co., Giza, Egypt) were used to determine the MDA, SOD, and GPx levels following the guidelines from the producer. The NO level was analyzed based on the nitrous acid diazotize sulfanilamide that forms when nitrite is present, and the product was linked with N-(1-naphthyl ethylenediamine). The resulting azo dye, brilliant reddish-purple, was measured at 540 nm. The MDA (an indicator of lipid peroxidation) level was measured using the thiobarbituric acid method based on thiobarbituric acid reacting with MDA to create thiobarbituric acid reactive species (pink-colored product), and the absorbance at 532 nm was measured. The SOD level was assayed based on its capacity to prevent the phenazine methosulfate-mediated reduction of nitro blue tetrazolium dye, and the absorbance was measured at 550 nm. The GPx level was estimated based on the conversion of NADPH into NADP+, and the absorbance was analyzed at 340 nm [73].

### 4.11. RT-qPCR

According to the manufacturer’s instructions, the total RNAs were isolated from the contralateral brain tissues using the Direct-zol TM RNA MiniPrep extraction kit and the TRIzol reagent. The isolated RNA’s purity and amount were assessed using a Nanodrop (UV–Vis Q5000 Spectrophotometer, Quawell Ltd., San Jose, CA, USA) Then, using the RevertAid First Strand cDNA Synthesis Kit (K1622; Thermo Fisher Scientific Inc., Rockford, IL, USA), the RNA was reverse transcribed to complementary DNA (cDNA). The mRNA levels of the target genes in each sample were determined using qPCR and the SYBR Green Master Mix (2x SensiFast TM SYBR, Bio-line). Table 1 presents the qPCR primers. Using β-actin as a reference, the expression of each gene was calculated using a 2^−ΔΔCt^ Ct method [74].

### 4.12. Histological Examination

The mice were euthanized at 72 h post-IR and/or after CBL treatment and transcardially perfused with 10 mL 0.9% ice-cold saline and 30 mL of 4% PFA [75]. The brain was extracted from the skull and fixed overnight at 4 °C with 4% PFA. Each fixed tissue was dehydrated via an upgraded ethanol series (40, 60, 70, 80, 90, and 100%, 60 min/each), cleared in xylene, and embedded and blocked in liquid paraffin wax. After that, serial coronal sections with a 5 μm thickness were obtained from each brain using a microtome, deparaffinized using xylene, progressively dehydrated with ethanol (100, 95, 70, and 50%, with 5 min/each), and rinsed in distilled water for five minutes. After hydration of all the tissues, each section was mounted on gelatin-coated slides and stained with hematoxylin and eosin (H&E) stain (Cat. No. DH0006; LEAGENE^®^) to identify the general histology and 0.1% cresyl violet stain (Nissl staining) (Cat. No. ab246817, Abcam, Cambridge, UK) for 10 min to determine the viable and nonviable neurons, as previously reported [76,77]. A light microscope with 400× magnification was used to capture each microscopic image.

### 4.13. Immunohistochemistry of GFAP and CD86

GFAP, an astrocyte marker, and CD68, a microglia marker, were immunostained using avidin–biotin immune peroxidase. Deparaffinized five μm slices were rehydrated, treated with 0.01 M citrate buffer, and incubated with 3% hydrogen peroxide (H_2_O_2_) for 30 min to decrease the endogenous peroxidase activity. After blocking with 10% normal goat serum in an antibody solution for 30 min, they were treated overnight at 4 °C with monoclonal mouse anti-GFAP (1:5000 diluted; Abcam, Cambridge, UK, Product Code: ab4674) and/or anti-CD68 (1:5000 diluted; Abcam, Cambridge, UK, Product Code: ab283316). After washing, the sections were treated with secondary antibodies, avidin-biotin complex, and hematoxylin counterstaining [78].

### 4.14. Morphometric Analysis

For the morphometric analysis, five mouse brains were randomly selected from each group, and five nonoverlapping microscopic fields per slide and three slides per animal were evaluated at 400× magnification. The photographs were taken and analyzed with the help of ImageJ. The mean number of viable and survival neurons with vesicular nuclei in the cerebral cortex, CA2, and dentate gyrus of the hippocampus, striatum, and thalamus, and the survival Purkinje cells in the cerebellum, were counted in the H&E-stained sections [79]. In addition, the mean density % of cresyl violet stain [80] and the area percentage of both the GFAP and CD68 immune expression were evaluated [81].

### 4.15. Data Analysis

The data were analyzed statistically using GraphPad Prism 8.0. A one-way analysis of variance was used to compare the parametric data (one-way ANOVA). Multiple comparisons of the means between groups were also performed using the Bonferroni test. *p* ≤ 0.05.was used as the threshold for statistical significance.

## 5. Conclusions

We conclude that CBL treatment post-ischemic-reperfusion injury improved neurological functional recovery, had anti-inflammatory and antioxidant properties, alleviated apoptotic neuronal death, and inhibited reactive microglial and astrocyte activation, resulting in neuroprotection after IR injury in the tDCAL/IR + CBL mice group. The TLRs/NF-kB/cytokines were suppressed, the Keap1/Nrf2/antioxidant signaling pathway was activated, VEGF was increased, and EDNRA expression was decreased. Our results show that CBL may improve neurologic function in mice following IR.

## 6. Study Limitations

This study’s limitations include the use of just male C57Bl6 mice, one dose of CBL, and the duration of the IR. Further studies should vary the gender of the mice, the strain of the mice, the dose of CBL, and the duration of the IR injury.

## Figures and Tables

**Table 1:** Primers used for gene expression.

Gene	GenBank Accession Number	Oligonucleotide Sequence	Annealing Temperature (°C)	Size (bp)
*IL-1β*	NM_008361.4	f5,-TGCCACCTTTTGACAGTGATG-3, r5,-TGATGTGCTGCTGCGAGATT-3,	60	138
*IL-6*	NM_001314054.1	f5,-GACAAAGCCAGAGTCCTTCAGA-3, r5,-TGTGACTCCAGCTTATCTCTTGG-3,	59	76
*TNF-α*	NM_001278601.1	f5,-ACTGAACTTCGGGGTGATCG-3, r5,-CCACTTGGTGGTTTGTGAGTG-3,	60	107
*IL10*	NM_010548.2	f5,-AGTGGAGCAGGTGAAGAGTG-3, r5,-TGGAGTCCAGCAGACTCAATAC-3,	58	160
*NF-κB*	AY388959.1	*F* 5,-CTGGCAAGCGTATCCCAAGA-3, *R*5,-TTCCGAAGTCGAACAGCCTC-3,	60	127
*TLR2*	NM_011905.3	*F* 5,-GCAGGAGATGTGTCCGCAAT-3, *R* 5,-AGAAGGAAACAGTCCGCACC-3,	62	111
*TLR4*	NM_021297.3	*F* 5,-GGACTCTGATCATGGCACTGT-3, *R* 5,-TCTTCAAGGGGTTGAAGCTC-3,	58	174
*Keap1*	NM_001110307.1	*F* 5,-GATGGGCAGGACCAGTTGAA-3, *R* 5,-CCGAGGACGTAGATCTTGCC-3,	60	134
*Nrf2*	NM_010902.4	*F* 5,-CCTCACCTCTGCTGCAAGTA-3, *R* 5,-AGCTCATAGTCCTTCTGTCGC-3,	59	205
*CAT*	NM_009804.2	*F* 5,-GCGGATTCCTGAGAGAGTGG-3, *R* 5,-TGTGGAGAATCGAACGGCAA-3,	59	145
*SOD3*	NM_011435.3	*F* 5,-GAGAAGATAGGCGACACGCA-3, *R* 5,-GAGAACCAAGCCGGTGATCT-3,	59	156
*GPX3*	NM_001329860.1	*F* 5,-CATCCTGCCTTCTGTCCCTG-3, *R* 5,-CGATGGTGAGGGCTCCATAC-3,	62	126
*VEGF*	NM_001025257.3	*F* 5,-TGAGACCCTGGTGGACATCT-3, *R* 5,-CACTCCAGGGCTTCATCGTT-3,	59	117
*EDNRA*	NM_010332.2	*F* 5,-TTGACCTCCCCATCAACGTG-3, *R* 5,-AGCACAGAGGTTCAAGACGG-3,	60	140
*β-actin*	AY618569.1	*F* 5,-GAGAGGGAAATCGTGCGTGA-3, *R* 5,-AACCGCTCGTTGCCAATAGT-3,	60	152

*IL-1β*; interleukin 1 beta, *IL-6*; Interleukin 6, *IL-10*; interleukin 10, *TNF-α*; tumor necrosis factor-alpha, *NF-κB*; nuclear factor kappa B, *TLR2*; Toll-like receptor 2, *TLR4*; Toll-like receptor 4, *Keap1*; Kelch-like ECH-associated protein 1, *Nrf2*; nuclear factor-erythroid factor 2-related factor 2, catalase; *CAT*; *SOD3*; superoxide dismutase 3, *VEGF*; vascular endothelial growth factor, *GPX3*; glutathione peroxidase 3, *EDNRA*; endothelin receptor type A, *β-actin*; beta-actin; *F*, forward; *R*, reverse.

## Data Availability

The datasets used and/or analyzed during the current study are available from the corresponding author upon reasonable request.

## References

[1] DeSai C., Hays Shapshak A. (2022). Cerebral Ischemia. StatPearls.

[2] Li W., Huang R., Shetty R.A., Thangthaeng N., Liu R., Chen Z., Sumien N., Rutledge M., Dillon G.H., Yuan F. (2013). Transient focal cerebral ischemia induces long-term cognitive function deficit in an experimental ischemic stroke model. Neurobiol. Dis..

[3] Lee N.T., Selan C., Chia J.S.J., Sturgeon S.A., Wright D.K., Zamani A., Pereira M., Nandurkar H.H., Sashindranath M. (2020). Characterization of a novel model of global forebrain ischaemia–reperfusion injury in mice and comparison with focal ischaemic and haemorrhagic stroke. Sci. Rep..

[4] Abumelha H.M., Alkhatib F., Alzahrani S., Abualnaja M., Alsaigh S., Alfaifi M.Y., Althagafi I., El-Metwaly N. (2021). Synthesis and characterization for pharmaceutical models from Co (II), Ni (II) and Cu (II)-thiophene complexes; apoptosis, various theoretical studies and pharmacophore modeling. J. Mol. Liq..

[5] Li Y., Zhang E., Yuan H. (2020). Cerebral carbon dioxide embolism after kidney cancer laparoscopic surgery with full neurological recovery: A case report. Medicine.

[6] Donnino M.W., Andersen L.W., Berg K.M., Reynolds J.C., Nolan J.P., Morley P.T., Lang E., Cocchi M.N., Xanthos T., Callaway C.W. (2015). Temperature management after cardiac arrest: An advisory statement by the advanced life support task force of the international liaison committee on resuscitation and the American Heart Association emergency cardiovascular care committee and the council on cardiopulmonary, critical care, Perioperative and Resuscitation. Circulation.

[7] Girotra S., Chan P.S., Bradley S.M. (2015). Post-resuscitation care following out-of-hospital and in-hospital cardiac arrest. Heart.

[8] Splichal Z., Jurajda M., Duris K. (2018). The Role of Inflammatory Response in Stroke Associated Programmed Cell Death. Curr. Neuropharmacol..

[9] Kang H.B., Kim G., Kim H., Han S.R., Chae D.J., Song H.-J., Kim D.W. (2013). Cerebrolysin Attenuates Astrocyte Activation Following Repetitive Mild Traumatic Brain Injury: Implications for Chronic Traumatic Encephalopathy. J. Life Sci..

[10] Sekhon M.S., Ainslie P.N., Griesdale D.E. (2017). Clinical pathophysiology of hypoxic ischemic brain injury after cardiac arrest: A “two-hit” model. Crit. Care.

[11] Hong D.K., Park Y.S., Woo J.S., Kim J.H., Beom J.H., Chung S.P., You J.S., Suh S.W. (2021). Transient Global Ischemia-Induced Brain Inflammatory Cascades Attenuated by Targeted Temperature Management. Int. J. Mol. Sci..

[12] Naumann T., Schnell O., Zhi Q., Kirsch M., Schubert K.O., Sendtner M., Hofmann H.D. (2003). Endogenous Ciliary Neurotrophic Factor Protects GABAergic, But Not Cholinergic, Septohippocam pal Neurons Following Fimbria-fornix Transection. Brain Pathol..

[13] Wronski R., Kronawetter S., Hutter-Paier B., Crailsheim K., Windisch M. (2000). A brain derived peptide preparation reduces the translation dependent loss of a cytoskeletal protein in primary cultured chicken neurons. Advances in Dementia Research.

[14] Bornstein N.M., Guekht A., Vester J., Heiss W.-D., Gusev E., Hömberg V., Rahlfs V.W., Bajenaru O., Popescu B.O., Muresanu D. (2018). Safety and efficacy of Cerebrolysin in early post-stroke recovery: A meta-analysis of nine randomized clinical trials. Neurol. Sci..

[15] Sadigh-Eteghad S., Geranmayeh M.H., Majdi A., Salehpour F., Mahmoudi J., Farhoudi M. (2018). Intranasal cerebrolysin improves cognitive function and structural synaptic plasticity in photothrombotic mouse model of medial prefrontal cortex ischemia. Neuropeptides.

[16] Huemer J., Plattner B., Planer N., Steiner H., Feucht M. (2016). Psychopathology in adolescents with TLE and FLE. Eur. J. Paediatr. Neurol..

[17] Yang G., Kitagawa K., Matsushita K., Mabuchi T., Yagita Y., Yanagihara T., Matsumoto M. (1997). C57BL/6 strain is most susceptible to cerebral ischemia following bilateral common carotid occlusion among seven mouse strains: Selective neuronal death in the murine transient forebrain ischemia. Brain Res..

[18] Ziganshina L.E., Abakumova T., Hoyle C.H. (2020). Cerebrolysin for acute ischaemic stroke. Cochrane Database Syst. Rev..

[19] Milot M.R., Plamondon H. (2009). Time-dependent effects of global cerebral ischemia on anxiety, locomotion, and habituation in rats. Behav. Brain Res..

[20] Wilson M., Staniforth A., Till R., das Nair R., Vesey P. (2014). The psychosocial outcomes of anoxic brain injury following cardiac arrest. Resuscitation.

[21] Neigh G.N., Karelina K., Glasper E.R., Bowers S.L., Zhang N., Popovich P.G., DeVries A.C. (2009). Anxiety after cardiac arrest/cardiopulmonary resuscitation: Exacerbated by stress and prevented by minocycline. Stroke.

[22] Guan X., Wang Y., Kai G., Zhao S., Huang T., Li Y., Xu Y., Zhang L., Pang T. (2019). Cerebrolysin Ameliorates Focal Cerebral Ischemia Injury Through Neuroinflammatory Inhibition via CREB/PGC-1α Pathway. Front. Pharmacol..

[23] Chamorro Á., Dirnagl U., Urra X., Planas A.M. (2016). Neuroprotection in acute stroke: Targeting excitotoxicity, oxidative and nitrosative stress, and inflammation. Lancet Neurol..

[24] Godinho J., de Oliveira R.M.W., de Sa-Nakanishi A.B., Bacarin C.C., Huzita C.H., Longhini R., Mello J.C.P., Nakamura C.V., Previdelli I.S., Ribeiro M.H.D.M. (2018). Ethyl-acetate fraction of Trichilia catigua restores long-term retrograde memory and reduces oxidative stress and inflammation after global cerebral ischemia in rats. Behav. Brain Res..

[25] Edel Hennessy E., Griffin W., Cunningham C. (2018). Cajal course on Neuroinflammation and how to study it Course directors. Glia.

[26] Jiang M., Liu X., Zhang D., Wang Y., Hu X., Xu F., Jin M., Cao F., Xu L. (2018). Celastrol treatment protects against acute ischemic stroke-induced brain injury by promoting an IL-33/ST2 axis-mediated microglia/macrophage M2 polarization. J. Neuroinflammation.

[27] Zhang B., Zhong Q., Chen X., Wu X., Sha R., Song G., Zhang C., Chen X. (2020). Neuroprotective effects of celastrol on transient global cerebral ischemia rats via regulating HMGB1/NF-κB signaling pathway. Front. Neurosci..

[28] Wine R.N., McPherson C.A., Harry G.J. (2009). IGF-1 and pAKT Signaling Promote Hippocampal CA1 Neuronal Survival Following Injury to Dentate Granule Cells. Neurotox. Res..

[29] Onken M., Berger S., Kristian T. (2012). Simple model of forebrain ischemia in mouse. J. Neurosci. Methods.

[30] Åberg N.D., Åberg D., Jood K., Nilsson M., Blomstrand C., Kuhn H.G., Svensson J., Jern C., Isgaard J. (2018). Altered levels of circulating insulin-like growth factor I (IGF-I) following ischemic stroke are associated with outcome—A prospective observational study. BMC Neurol..

[31] Obaid R. (2021). Synthesis and biological evaluation of some new imidazo [1, 2-c] pyrimido [5, 4-e] pyrimidin-5-amine derivatives. J. Umm Al-Qura Univ. Appl. Sci..

[32] El-Beeh M.E., El-Badawi A.A., Amin A.H., Qari S.H., Ramadan M.F., Filfilan W.M., El-Sayyad H.I. (2022). Anti-aging trait of whey protein against brain damage of senile rats. J. Umm Al-Qura Univ. Appl. Sci..

[33] Sage J., Van Uitert R.L., Duffy T. (1984). Early changes in blood brain barrier permeability to small molecules after transient cerebral ischemia. Stroke.

[34] Lamanna J.C., Kaiserman-Abramof I., Xu K., Daugherty S., Chávez J.C., Pichiule P. (2001). Acute and Delayed Effects of Transient Global Cerebral Ischemia on Rat Brain Capillary Endothelial Cells In Vivo. Ischemic Blood Flow in the Brain.

[35] Zhao X., Li S., Mo Y., Li R., Huang S., Zhang A., Ni X., Dai Q., Wang J. (2021). DCA Protects against Oxidation Injury Attributed to Cerebral Ischemia-Reperfusion by Regulating Glycolysis through PDK2-PDH-Nrf2 Axis. Oxidative Med. Cell. Longev..

[36] Chen H., Yoshioka H., Kim G.S., Jung J.E., Okami N., Sakata H., Maier C.M., Narasimhan P., Goeders C.E., Chan P.H. (2011). Oxidative Stress in Ischemic Brain Damage: Mechanisms of Cell Death and Potential Molecular Targets for Neuroprotection. Antioxid. Redox Signal..

[37] Sharma H.S., Muresanu D.F., Ozkizilcik A., Sahib S., Tian Z.R., Lafuente J.V., Nozari A., Feng L., Buzoianu A.D., Menon P.K. (2021). Superior antioxidant and anti-ischemic neuroprotective effects of cerebrolysin in heat stroke following intoxication of engineered metal Ag and Cu nanoparticles: A comparative biochemical and physiological study with other stroke therapies. Prog. Brain Res..

[38] Liu G., Zhang L., Zhao Y. (2010). Modulation of immune responses through direct activation of Toll-like receptors to T cells. Clin. Exp. Immunol..

[39] Vabulas R.M., Ahmad-Nejad P., da Costa C., Miethke T., Kirschning C.J., Häcker H., Wagner H. (2001). Endocytosed HSP60s Use Toll-like Receptor 2 (TLR2) and TLR4 to Activate the Toll/Interleukin-1 Receptor Signaling Pathway in Innate Immune Cells. J. Biol. Chem..

[40] Shan R., Zhou H., Liu X., Su G., Liu G., Zhang X., Sun C., Yu Z., Zhan L., Huang Z. (2021). Neuroprotective effects of four different fluids on cerebral ischaemia/reperfusion injury in rats through stabilization of the blood–brain barrier. Eur. J. Neurosci..

[41] Cores Á., Piquero M., Villacampa M., León R., Menéndez J.C. (2020). NRF2 Regulation Processes as a Source of Potential Drug Targets against Neurodegenerative Diseases. Biomolecules.

[42] Tebay L.E., Robertson H., Durant S.T., Vitale S.R., Penning T.M., Dinkova-Kostova A.T., Hayes J.D. (2015). Mechanisms of activation of the transcription factor Nrf2 by redox stressors, nutrient cues, and energy status and the pathways through which it attenuates degenerative disease. Free Radic. Biol. Med..

[43] Wardlaw J., Sandercock P., Dennis M., Starr J. (2003). Is Breakdown of the Blood-Brain Barrier Responsible for Lacunar Stroke, Leukoaraiosis, and Dementia?. Stroke.

[44] Pinard E., Nallet H., MacKenzie E.T., Seylaz J., Roussel S. (2002). Penumbral microcirculatory changes associated with peri-infarct depolarizations in the rat. Stroke.

[45] Hermann D.M., Zechariah A. (2009). Implications of Vascular Endothelial Growth Factor for Postischemic Neurovascular Remodeling. J. Cereb. Blood Flow Metab..

[46] Marghani B.H., Fehaid A., Ateya A.I., Ezz M.A., Saleh R.M. (2022). Photothermal therapeutic potency of plasmonic silver nanoparticles for apoptosis and anti-angiogenesis in testosterone induced benign prostate hyperplasia in rats. Life Sci..

[47] Stanimirovic D., Mccarron R., Bertrand N., Spatz M. (1993). Endothelins Release 51Cr from Cultured Human Cerebromicrovascular Endothelium. Biochem. Biophys. Res. Commun..

[48] Sharma H.S., Zimmermann-Meinzingen S., Johanson C.E. (2010). Cerebrolysin reduces blood-cerebrospinal fluid barrier permeability change, brain pathology, and functional deficits following traumatic brain injury in the rat. Ann. N. Y. Acad. Sci..

[49] Irmak M.K., Fadillioglu E., Sogut S., Erdogan H., Gulec M., Ozer M., Yagmurca M., Gozukara M.E. (2003). Effects of caffeic acid phenethyl ester and alpha-tocopherol on reperfusion injury in rat brain. Cell Biochem. Funct..

[50] Chan P.H. (2001). Reactive Oxygen Radicals in Signaling and Damage in the Ischemic Brain. J. Cereb. Blood Flow Metab..

[51] El Sayed N.S., Kandil E.A., Ghoneum M.H. (2021). Probiotics Fermentation Technology, a Novel Kefir Product, Ameliorates Cognitive Impairment in Streptozotocin-Induced Sporadic Alzheimer’s Disease in Mice. Oxidative Med. Cell. Longev..

[52] Türeyen K., Vemuganti R., Sailor K.A., Dempsey R.J. (2004). Infarct volume quantification in mouse focal cerebral ischemia: A comparison of triphenyltetrazolium chloride and cresyl violet staining techniques. J. Neurosci. Methods.

[53] Zhang L., Chopp M., Zhang Z.G. (2013). Abstract WMP36: The Sonic Hedgehog Signaling Pathway Mediates Cerebrolysin-Improved Neurological Functions after Stroke. Stroke.

[54] Ubhi K., Rockenstein E., Doppler E., Mante M., Adame A., Patrick C., Trejo M., Crews L., Paulino A., Moessler H. (2009). Neurofibrillary and neurodegenerative pathology in APP-transgenic mice injected with AAV2-mutant TAU: Neuroprotective effects of Cerebrolysin. Acta Neuropathol..

[55] Guzmán D.C., Brizuela N.O., Álvarez R.G., García E.H., Mejía G.B., Olguín H.J. (2009). Cerebrolysin and morphine decrease glutathione and 5-hydroxyindole acetic acid levels in fasted rat brain. Biomed. Pharmacother..

[56] Gutmann B., Hutter-Paier B., Skofitsch G., Windisch M., Gmeinbauer R. (2002). In vitro models of brain ischemia: The peptidergic drug cerebrolysin protects cultured chick cortical neurons from cell death. Neurotox. Res..

[57] Jha M.K., Jo M., Kim J.-H., Suk K. (2019). Microglia-Astrocyte Crosstalk: An Intimate Molecular Conversation. Neuroscientist.

[58] Liddelow S.A., Barres B.A. (2017). Reactive Astrocytes: Production, Function, and Therapeutic Potential. Immunity.

[59] Pekny M., Nilsson M. (2005). Astrocyte activation and reactive gliosis. Glia.

[60] Strużyńska L., Dąbrowska-Bouta B., Koza K., Sulkowski G. (2007). Inflammation-Like Glial Response in Lead-Exposed Immature Rat Brain. Toxicol. Sci..

[61] Kingham P.J., Cuzner M.L., Pocock J.M. (1999). Apoptotic Pathways Mobilized in Microglia and Neurones as a Consequence of Chromogranin A-Induced Microglial Activation. J. Neurochem..

[62] Schnieder T.P., Trencevska I., Rosoklija G., Stankov A., Mann J.J., Smiley J., Dwork A.J. (2014). Microglia of prefrontal white matter in suicide. J. Neuropathol. Exp. Neurol..

[63] Ma Y., Wang J., Wang Y., Yang G.-Y. (2017). The biphasic function of microglia in ischemic stroke. Prog. Neurobiol..

[64] Annunziato L., Boscia F., Pignataro G. (2013). Ionic transporter activity in astrocytes, microglia, and oligodendrocytes during brain ischemia. J. Cereb. Blood Flow Metab..

[65] Liu L.-R., Liu J.-C., Bao J.-S., Bai Q.-Q., Wang G.-Q. (2020). Interaction of Microglia and Astrocytes in the Neurovascular Unit. Front. Immunol..

[66] Barakat W., Safwet N., El-Maraghy N.N., Zakaria M.N. (2014). Candesartan and glycyrrhizin ameliorate ischemic brain damage through downregulation of the TLR signaling cascade. Eur. J. Pharmacol..

[67] Kane M.J., Angoa-Pérez M., Briggs D.I., Viano D.C., Kreipke C.W., Kuhn D.M. (2012). A mouse model of human repetitive mild traumatic brain injury. J. Neurosci. Methods.

[68] Caston J., Jones N., Stelz T. (1995). Role of Preoperative and Postoperative Sensorimotor Training on Restoration of the Equilibrium Behavior in Adult Mice Following Cerebellectomy. Neurobiol. Learn. Mem..

[69] Rogers D.C., Campbell C.A., Stretton J.L., Mackay K. (1997). Correlation Between Motor Impairment and Infarct Volume After Permanent and Transient Middle Cerebral Artery Occlusion in the Rat. Stroke.

[70] Archer J. (1973). Tests for emotionality in rats and mice: A review. Anim. Behav..

[71] Menard J., Treit D. (1999). Effects of centrally administered anxiolytic compounds in animal models of anxiety. Neurosci. Biobehav. Rev..

[72] Keep R.F., Hua Y., Xi G. (2012). Brain water content: A misunderstood measurement?. Transl. Stroke Res..

[73] Marghani B.H., El-Adl M., Ateya A.I., Othman B.H., Ghamry H.I., Shukry M., Soliman M.M., Rizk M.A. (2022). The Potential Protective Effect and Underlying Mechanisms of Physiological Unconjugated Hyperbilirubinemia Mediated by UGT1A1 Antisense Oligonucleotide Therapy in a Mouse Model of Cyclosporine A-Induced Chronic Kidney Disease. Metabolites.

[74] Pfaffl M. (2001). A new mathematical model for relative quantification in real-time RT-PCR. Nucleic Acids Res..

[75] Yin W., Badr A.E., Mychaskiw G., Zhang J.H. (2002). Down regulation of COX-2 is involved in hyperbaric oxygen treatment in a rat transient focal cerebral ischemia model. Brain Res..

[76] Horobin R.W. (2018). Theory of histological staining. Bancroft’s Theory and Practice of Histological Techniques.

[77] Jones M.L. (2015). Histotechnology: A Self Instructional Text. J. Histotechnol..

[78] Petrosyan K., Tamayo R., Joseph D. (2002). Sensitivity of a novel biotin-free detection reagent (Powervision+™) for immunohistochemistry. J. Histotechnol..

[79] Lehr C.A., Tan C.S., Ysseldyke J. (2009). Alternative schools: A synthesis of state-level policy and research. Remedial Spec. Educ..

[80] Elsayed S., El-Habeby M., El-Sherif N., Al-Gholam M. (2021). Influence of global cerebral ischemia/reperfusion injury on rat dentate gyrus and the possible protective effect of beetroot (*Beta vulgaris* L.) extract. Eur. J. Anat..

[81] Karthik L., Kumar G., Keswani T., Bhattacharyya A., Chandar S.S., Rao K.V.B. (2014). Protease Inhibitors from Marine Actinobacteria as a Potential Source for Antimalarial Compound. PLoS ONE.

